# Investigations Concerning the Residence Time Distribution of Twin-Screw-Extrusion Processes as Indicator for Inherent Mixing

**DOI:** 10.3390/pharmaceutics10040207

**Published:** 2018-10-26

**Authors:** Jens Wesholowski, Andreas Berghaus, Markus Thommes

**Affiliations:** 1 Institute of Solids Process Engineering, TU Dortmund University, 44227 Dortmund, Germany; jens.wesholowski@tu-dortmund.de; 2 COLVISTEC AG, 12489 Berlin, Germany; a.berghaus@colvistec.de

**Keywords:** modeling, process control, residence time, twin-dispersion-model, twin-screw-extrusion

## Abstract

Over recent years Twin-Screw-Extrusion (TSE) has been established as a platform technology for pharmaceutical manufacturing. Compared to other continuous operation, one of the major benefits of this method is the combination of several unit operations within one apparatus. Several of these are linked to the Residence Time Distribution (RTD), which is typically expressed by the residence time density function. One relevant aspect for pharmaceutical processes is the mixing capacity, which is represented by the width of this distribution. In the frame of this study the influence of the mass flow, the temperature and the screw-barrel clearance were investigated for a constant barrel load (specific feed load, SFL). While the total mass flow as well as the external screw diameter affected the mixing performance, the barrel temperature had no influence for the investigated range. The determined results were additionally evaluated with respect to a fit to the Twin-Dispersion-Model (TDM). This model is based on the superimposition of two mixing functions. The correlations between varied process parameters and the obtained characteristic model parameters proved this general physical view on extrusion.

## 1. Introduction

Over the last decade, continuous manufacturing techniques have been focused on pharmaceutical production, since these offer several benefits for the implementation of the Quality-by-Design (QbD) concepts [1,2,3]. In general, this approach is driven by the reduction of product quality fluctuations by sufficient control strategies.

An emerging technology in this context is Twin-Screw-Extrusion (TSE) [4], which is generally applied for bioavailability enhancement [5] as well as the preparation of different solid dosage forms such as films [6] or implants [7]. Additional advantages compared to the continuous operation are a solvent-free and cost-efficient process [8] as well as a modular set-up of the screw configuration [9]. Due to the incorporation of different element types, e.g., conveying or kneading, also several unit operations can be combined within one processing step such as melting, dissolving reacting or degassing [10,11].

A critical sub-process for the manufacturing of pharmaceuticals is the mixing of the material. The correlation to process and construction parameters and the effect on the product quality has been frequently investigated for TSE [12,13,14,15]. However, the mixing potential of an extrusion process is correlated with the Residence Time Distribution (RTD). This parameter is normally expressed by the residence time density function E and can be determined by a Dirac-impulse experimentally [16]. The onset represents the minimum mixing time and is crucial for dissolving mechanisms [17]. The width of the distribution symbolizes the total mixing capacity [18]. This is the potential to balance out local concentration gradients of single components. This type of mixing is classified as distributive mixing and has to be considered separately from dispersive mixing [19], which is the ability to even particle sizes during processing. Overall, the RTD is a suitable measure in terms of material traceability for TSE and continuous manufacturing in general [20]. 

The insights regarding the RTD can be used for process design and control by a suitable RTD model [21,22]. The characteristic model parameters function as surrogate parameter for the current process conditions. The Axial-Dispersion-model or the Tanks-in-Series-model are not capable to catch the offset of a RTD [23] in contrast to the Two-Compartment-model [24]. The results for the Z-function [25] were on the same level. However, the physical meaning of the characteristic parameters is not defined. In general, the superiority of considering two mixing mechanism driving TSE for a RTD fit was further demonstrated within [23]. Based on the results the TDM was developed. The parameters of this approach directly reflect the flow conditions related to both mixing mechanisms, which is the major benefit of this model in comparison to the Two-Compartment-model. 

The aim of this study is the investigation of the physical hypothesis that TSE is driven by two separate mixing processes. For the experimental determination of the RTD an inline UV/Vis spectrophotometer is used. During the experiments, process and machine parameters are varied to investigate the corresponding effect on the RTD. This can be identified by the course of the function. This type of process related response is additionally evaluated with respect to fitted characteristic model parameters of the TDM [23].

## 2. Materials and Methods

### 2.1. Hot-Melt-Extrusion on a Co-Rotating Twin-Screw-Extruder

The polymer polyvinylpyrrolidone vinylacetate (copovidone; Kollidon VA 64, BASF, Ludwigshafem, Germany) was used as model substance and dosed by a gravimetric feeder (KT20, K-Tron, Niederlenz, Switzerland). The recommended temperature for the extrusion of this material is in the range of 155 to 200 °C according to [26]. For the RTD determination quinine dihydrochloride with a melting point above 230 °C (Caesar & Loretz, Hilden, Germany) served as tracer material. For all experiments 1 g was used as initial concentration per 400 g min^−1^ of material flow of the model substance. The tracer was added as Dirac-impulse in steady state into the hopper of the co-rotating Twin-Screw-Extruder (Leistritz ZSE 27 Maxx, Nurenberg, Germany). The applied screw configuration is commonly used within literature [24] and consisted of conveying (GFA) and kneading elements (KB). At the feed port conveying elements with an especially large free volume (GFF) were implemented. The element marks are further explained in [27].

A first series of experiments was performed for a constant specific feed load (SFL) at three different combinations of screw speed *n* and mass flow of the polymer $\dot{m}$ (see Table 1), while the temperature barrel profile was not varied. 

The SFL symbolizes the load inside the extruder and correlates directly to the ratio of mass flow to screw speed (1), if the material density ρ as well as the barrel diameter *d* remain constant.

(1) $$\mathrm{SFL}=\frac{\dot{m}}{n\;\rho \;d^{3}}$$

**Figure 1:**
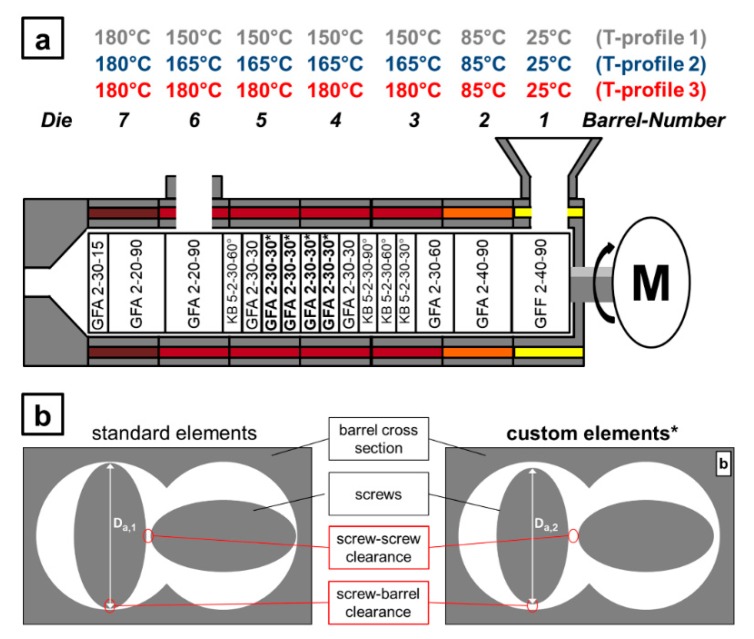
(**a**) Used screw configuration and varied barrel temperature profiles for the extrusion experiments. (**b**) Display of standard (left) and custom (right) screw elements within the barrel cross section. Diameter D_a,1_ is larger than D_a,2_ while the barrel diameter remains constant.

In a second experimental series the screw speed was 100 rpm at a mass flow of 2.4 kg/h and the barrel temperature was varied for specific sections from 150 to 180 °C at three levels (see Figure 1a).

For a third experimental series the previous ones were repeated for a different screw configuration, with incorporated elements at the wear limit implemented at the marked position (*) within Figure 1. Therefore, the outer diameter of these custom designed elements was reduced (Figure 1b) and the clearance between screws and barrel as well as from screw to screw increased by this. This set-up simulated the performance after long-term use.

The absolute reduction from the standard to the custom designed elements was 1.1 mm to an external screw diameter of 27.2 mm. In comparison to the standard nominal outer diameter of 28.3 mm this is negligible regarding the effect on the SFL in this section of the screw configuration. All extrusion experiments were carried out in triplicate.

### 2.2. Inline Determination of the RTD

The RTD was determined by the marker concentration with a UV/Vis spectrophotometer (InSpectro X, ColVisTec, Germany) at a measurement frequency of 0.9 Hz over the time *t*. Two measurement probes (TPMP, ColVisTec, Germany) were implemented inline in the flow channel of the extrusion die. The determined signal in form of the ratio of light intensity *I* to the basic light intensity *I*_0_ as a function of the wavelength λ was evaluated in two steps. First the detected signal was converted into an absorbance value A in accordance to the Lambert-Beer law and integrated for the active spectra range (300 to 800 nm) of the used marker (2). After a baseline correction the signal values was nominated by the integral of the overall RTD in a second step and the residence time density function E was derived (3).

(2) $$A_{300–800\mathrm{nm}}\left(t\right)=\;\int_{300\mathrm{nm}}^{800\mathrm{nm}}\left(-\log_{10}\frac{I\left(\lambda ,t\right)}{I_{0}\left(\lambda \right)}\right)d\lambda$$

(3) $$E\left(t\right)=\;\frac{A_{300–800\mathrm{nm}}\left(t\right)}{\int A_{300–800\mathrm{nm}}\left(t\right)dt}$$

The course of the determined distribution can be represented by the characteristic quantiles *t_i_*, which symbolize in this case the exit time for *i* % of the marker and is calculated based on the cumulative residence time density function (4).

(4) $$\frac{i}{100}=\int_{0s}^{t_{i}}E\left(t\right)dt$$

## 3. Results

### 3.1. Effect of Mass Flow for Constant Barrel Load and Temperature Profile

**Figure 2:**
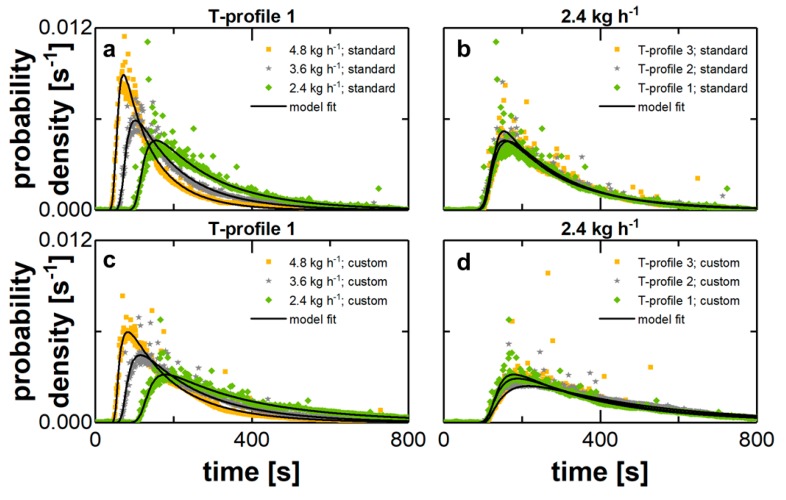
Effect of investigated parameters on the RTD. Determined residence time density functions for each parameter set are represented by the run, which symbolizes the average of the three repetitions: (**a**) variation of mass flow for standard elements; (**b**) variation of temperature profile for standard elements; (**c**) variation of mass flow for customized elements; (**d**) variation of temperature profile for customized elements.

The influence of the total mass flow on the RTD was tested for a constant load of the extruder with an SFL of 0.014. During process development, optimization in terms of increasing the efficiency is related to maximizing the load in a first step. In a second step the throughput is usually maximized while the load is kept constant. This procedure does affect the RTD directly and was investigated for three levels of parameter sets ($\dot{m}$, *n*) and for a standard screw configuration. The results are presented in Figure 2 for each parameter set by the different symbols by a representative run of the three repetitions, which symbolizes the mean average. Overall the results of all runs for one parameter set and screw configuration were in a good agreement in each case (see Table 2).

For each measurement, a characteristic function is found, which consist of a steep ascending up to the peak and an in comparison decelerated kinetic for the descending flank. So overall the distribution is asymmetric in all cases, which has been reported for HME [24,25]. However, the discrete time of the onset, peak and offset are shifted to smaller values for an increasing mass flow, since the filled volume of the extruder respectively the SFL remained constant. The overall distribution of the signal impulse is tighter, which is indicated by the maximum value in the peak region as well as the slope of the descending flank. These are higher respectively steeper for a greater total mass flow. In conclusion, the distributive mixing performance is higher for a smaller throughput at a constant extruder load. At the same time, this increases the risk of thermal material degradation due to a time extended temperature stress applied to the material for a constant barrel temperature profile.

### 3.2. Effect of Temperature Profile for Constant SFL and Total Mass Flow

The processing temperature during Hot-Melt-Extrusion is relevant regarding the dissolution kinetics of a pharmaceutical in a polymer melt. Also, the RTD is affected by this due to a direct impact on the viscosity and the related flow mechanisms. 

For the standard screw configuration, the processing temperature was changed by the barrel temperature profile in certain sections of the processing zone (see Figure 1). The variations included three levels from 150 to 165 °C and finally to 180 °C in this series of experiments. The remaining process parameters were kept constant. The experimental results in terms of the measured RTD are shown in Figure 2b for a representative repetition of each experiments, which were executed in triplicate.

The variation of the barrel temperature in some specific processing sections had a minor effect on the RTD. Onset, peak, and offset are located at the same time, while the course of the distribution is similar. Potentially, the increase of the barrel temperature by 30 K does not automatically lead to an increase of the material temperature inside the barrel by the same, since this correlation is not necessarily linear. The heating of the material during TSE is also affected by e.g., shearing. When this heating mechanisms is dominant, the actual melt temperature can be higher than the barrel temperature and an adjustment of this would have a minor effect. 

In conclusion, a deviation of the barrel temperature up to 30 K higher than the set value in the tested processing sections was not relevant for the residence time of the process with respect to the tested set-up. This is important for the precise control of dissolution and degradation mechanisms and in terms of process monitoring.

### 3.3. Effect of Clearance for Constant SFL and Mass Flow or Temperature Profile

In addition to the previous investigations, the effect of the clearance between screw and barrel was studied. This parameter should increase during long-term use of the equipment due to material wear. Custom designed elements with a reduced outer diameter at the wear limit were incorporated in a section of the screw configuration. For this set-up both experimental series related to the influence of the throughput and the barrel temperature on the RTD were repeated.

For an increasing throughput, the onset and peak are located at the same time (see Figure 2a,c), while the discharge kinetic is elongated using conveying elements with a reduced outer screw diameter. The slope of the descending flank is reduced, the maximum value of the peak is lower, and the retention time is longer. Also, for the temperature profile variations the extended clearance emphasized distributive material mixing related to a broader RTD, which is probably related to a mass flow over the screw tips. This leads to a flattening of the RTD especially for the discharge kinetic. This mixing process is superimposed by a general material transport close to plug flow, which is linked to the ascending flank. The narrowness of this section indicates small axial dispersion for a part of the material. The direct respond to a higher throughput is a shift to smaller residence times. So, the two kinetics represented by the RTD are defined by two different mixing respectively transport processes.

The custom designed elements, lead to a broadening of the overall RTD, while the peak was flattened for both investigated process parameters. This correlation is expressed by the characteristic quantiles *t*_10_, *t*_50_ and *t*_90_. These are increasing for the elements with a reduced diameter (Table 2), since the area under the curve is less focused around the peak and more spread. The calculated results for the quantiles also highlight the sufficient repeatability of the individual extrusion experiments due to coefficients of variation smaller than 8%.

In conclusion, the RTD is a potential measure to identify the degree of wear of screw elements, since the distribution is stretched for a larger clearance. This will also directly affect the product quality, since the duration of thermal and mechanical stress applied to the processed material is extended in this case. However, to characterize the degree of wear by the RTD further experiments and a quantitative analysis must be performed.

### 3.4. Representation of Investigated Effects by RTD Model Parameters

The superimposition of two mixing process as basis for transport phenomena in TSE is the physical background of the Twin-Dispersion-Model (TDM) [23]. The single mixing processes are represented by the residence time density function according to the Axial-Dispersion-Model (ADM) [28,29]. Here the Bodenstein-number Bo symbolizes the ratio of dispersion to transport each in axial direction, while increasing values represented a shift to plug flow. Therefore, two approaches for Bo ≤ 100 (5) and Bo > 100 (6) must be distinguished. Overall, the TDM considers two mean residence times $\bar{t}$.

(5) $$E\left(t\right)=\frac{1}{2\cdot \bar{t}}{\left(\frac{Bo}{\pi \;\frac{t}{\bar{t}}}\right)}^{0.5}e^{-\frac{Bo\;{\left(1-\frac{t}{\bar{t}}\right)}^{2}}{4\;\frac{t}{\bar{t}}}}$$

(6) $$E\left(t\right)=\frac{1}{2\cdot \bar{t}}{\left(\frac{Bo}{\pi }\right)}^{0.5}e^{-\frac{Bo\;{\left(1-\frac{t}{\bar{t}}\right)}^{2}}{4}}$$

The TDM proposes the convolution of two of these functions (7). Due to a nomination by the overall function integral as area under the curve AUC the residence time density function E for the TDM is derived. For non-nominated data sets c_0_ is considered to be scaling parameter for the overall determined signal concentration c. In theory Bo_1_ symbolizes the material flow within the die channel and material exchange over the screw tips by Bo_2_. The duration of each mechanisms is represented by ${\bar{t}}_{1}$ and ${\bar{t}}_{2}$.

(7) $$c\left(t\right)=\;\frac{c_{0}}{\mathrm{AUC}}\overset{\infty }{\underset{-\infty }{\int }}E{\left(s,\;{\bar{t}}_{1},\;{\mathrm{Bo}}_{1}\right)}_{\mathrm{ADM}}\;E{\left(t-s,\;{\bar{t}}_{2},\;{\mathrm{Bo}}_{2}\right)}_{\mathrm{ADM}}ds=c_{0}\;E{\left(t\right)}_{\mathrm{TDM}}$$

The TDM was fitted to the extrusion data sets by Python 2.7.0 and least-square method was used to obtain the characteristic model parameters. The overall sufficient agreement between experimental and fitted values is highlighted within Figure 2. The model fit is symbolized for each representative run by the lines.

**Figure 3:**
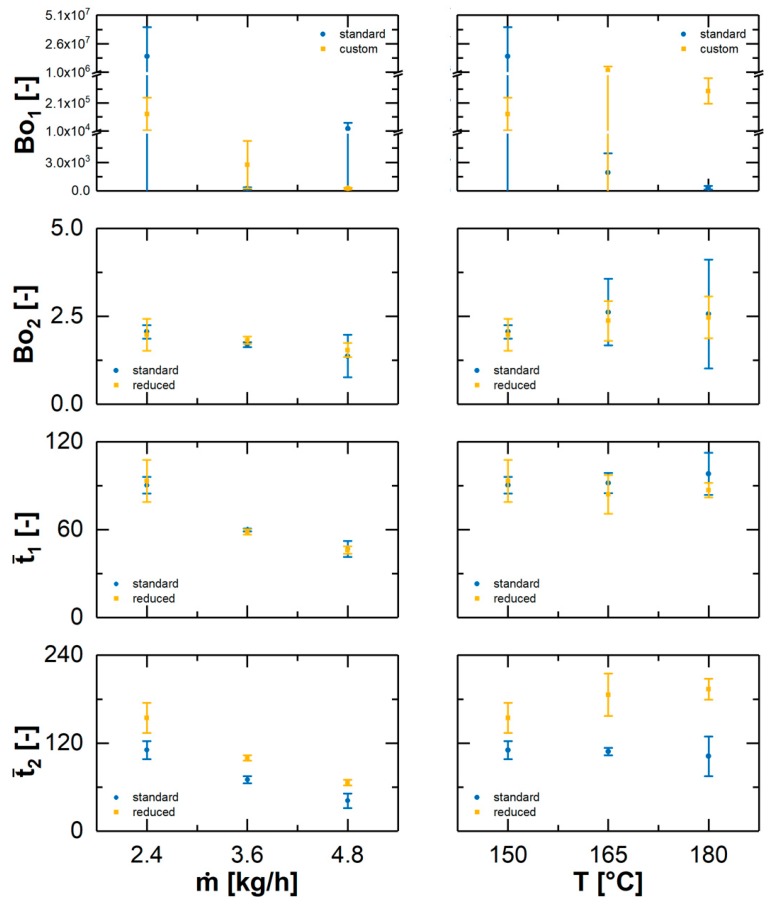
Obtained characteristic model parameters in comparison for a screw configuration with standard element and custom designed elements with a reduced outer diameter for an enlarged clearance. The effect of the total mass flow and of the barrel temperature profile is presented separately (av ± s, *n* = 3).

The determined model parameters are shown in Figure 3. An effect for Bo_1_ based on the standard deviation for these parameters cannot be reported for the investigated variation of the SFL, barrel temperature or the gap width. This parameter mainly defines the ascending flank of the RTD. Since this is typically steep, values for Bo_1_ are frequently higher than 100. In this range the basic function is extremely sensitive towards fluctuations in the experimental data sets. Therefore, the coefficient of variation for this model parameter is usually exceeding the coefficient of variation of the raw experimental data, e.g., of the quantiles, and finally the results cannot be evaluated meaningful.

The parameter Bo_2_ reflects in the model theory the material mixing by a flow over the screw tips. Therefore, the response to the different parameter variations is as expected. In tendency a higher throughput leads to smaller parameter values, while an increasing temperature has the opposite effect. Also, the enlargement of the clearance is negligible concerning this parameter. In general, the obtained values are all on the same level and the absolute deviation between different parameter sets is rather small. These overall inferior effects might be related to the fact, that the obtained Bo-values represent nearly ideal axial mixing.

The parameter ${\bar{t}}_{1}$ symbolizes the duration of the main material transport. Therefore, the obtained values are reduced for a higher total mass flow, since according to the constant SFL during these experiments the filled volume remained theoretically the same. The variation of the clearance as well as the temperature did not lead to a change of the duration time, which is in a good agreement with the experimental data. In conclusion, these are not variables affecting the axial transport of the material during extrusion.

Finally, the parameter ${\bar{t}}_{2}$ is estimated to be linked to the overall duration time for the second mixing process. Based on the model theory this is correlating with the material flow over the screw tips respectively trough the clearance. The presented results support this hypothesis, since the ${\bar{t}}_{2}$-values are shrinking for an increasing total mass flow at a constant load of the extruder. The transport velocity of the material is enhanced leading to higher drag forces, while the increased screw speed at a constant fill level leads to higher shear forces at the screw tips. Therefore, the material should be scraped off faster. In addition, for a larger clearance the duration times for the identical process parameters are higher in each case. However, for both screw configurations the ${\bar{t}}_{2}$-values are shrinking for an increasing total mass flow at a constant SFL. In contrast, for the variation of the temperature profile with respect to the error bars there are no significant differences for ${\bar{t}}_{2}$ for the individual screw configuration. This is in a good agreement with the obtained experimental data. In comparison, the observed tendencies of the standard screw elements to the customized screw elements are antithetical and the values for the time parameter are shifted to a higher level for a larger clearance. The enlarged gap size favors a potential flow of material over the screws, which leads to a longer duration of this mechanism. Thereby the ${\bar{t}}_{2}$-parameter values increases slightly and could be used as equipment performance indicator in industrial procedures. However, further quantitative analysis must be conducted to investigate this relation.

## 4. Conclusions

Within this study the influence of the mass flow and the barrel temperature for a constant SFL as process parameters and the clearance as machine parameter were investigated regarding the corresponding effect on the RTD. The mixing capacity was reduced for a constant SFL for higher throughputs due to a narrowing of the distribution, while a larger clearance emphasized this sub-process. Therefore, the RTD is also a suitable measure for the wear of the screw elements. In contrast, the barrel temperature had a minor effect. These observations were approved by obtained model parameters for a fit of the experimental data sets to a model. The mixing related to material flow over the screw tips is represented by the Bo_2_-parameter and the duration of this by the parameter ${\bar{t}}_{2}$. The mixing capacity was enhanced by the clearance and the barrel temperature in terms of the viscosity. Additionally, these respond to a higher total mass flow for a constant SFL by a reduction of the overall mixing potential. 

In conclusion, the used Twin-Dispersion-Model is suitable to represent the two mixing processes occurring during extrusion. The model parameters were correlated with process and operating parameters and the effect on the RTD was revealed.

## Figures and Tables

**Table 1:** Process parameters for the two different sets of extrusion experiments.

$\dot{m}$ [kg h^−1^]	*n* [min^−1^]	SFL [-]	Barrel Temperature Profile
2.4	100	0.014	T-profile 1
3.6	150	0.014	T-profile 1
4.8	200	0.014	T-profile 1
2.4	100	0.014	T-profile 1
2.4	100	0.014	T-profile 2
2.4	100	0.014	T-profile 3

**Table 2:** Characteristic quantiles represented as mean value ± standard deviation (*n* = 3) of the determined RTD for a constant SFL = 0.014.

$\dot{m}$ [kg h^−1^]	*n* [min^−1^]	*T*_Barrel 3–6_ [°C]	Screw Elements	*t*_10_ [s]	*t*_50_ [s]	*t*_90_ [s]
2.4	100	150	standard	140.4 ± 2.7	247.4 ± 7.6	486.4 ± 12.5
			reduced	163.4 ± 6.5	314.4 ± 9.5	664.8 ± 27.0
3.6	150	150	standard	90.2 ± 1.3	168.1 ± 6.4	418.6 ± 7.6
			reduced	102.7 ± 0.8	207.8 ± 2.3	483.1 ± 13.0
4.8	200	150	standard	63.9 ± 1.3	120.0 ± 5.2	317.3 ± 35.7
			reduced	74.5 ± 0.2	152.0 ± 4.2	371.6 ± 32.3
2.4	100	165	standard	143.3 ± 1.9	238.1 ± 13.3	454.3 ± 12.2
			reduced	169.3 ± 3.2	335.6 ± 10.9	658.1 ± 13.7
2.4	100	180	standard	149.4 ± 4.5	240.4 ± 9.2	439.1 ± 45.5
			reduced	177.9 ± 0.2	346.7 ± 31.3	698.2 ± 76.4

## Abbreviations

A	absorbance of light as function of wavelength	[-]
AUC	area under the function curve/function integral	[-]
Bo	Bodenstein-number	[-]
*c*_0_	scaling parameter	[*]
*c*	signal concentration	[*]
*d*	external screw diameter	[m]
E	residence time density function	[s^−1^]
*I*	transmitted light intensity as function of wavelength	[-]
*I*_0_	basic light intensity as function of wavelength	[-]
$\dot{m}$	total mass flow of powder inlet	[kg s^−1^]
*n*	screw speed	[kg]
SFL	specific feed load	[-]
*t*	time	[s]
$t_{i}$	time to a quantile value of i%	[s]
$\bar{t}$	mean residence time	[s]
*T*	temperature	[°C]
λ	wavelength	[nm]
ρ	density	[kg m^−3^]
* unit depends on measured signal		
